# Enhanced Bioavailability of Nadolol via Cinnamon Oil-Based Microemulsions: Formulation, Characterization, Cell Culture, and *In Situ* Intestinal Perfusion Studies

**DOI:** 10.1021/acsomega.6c02057

**Published:** 2026-07-29

**Authors:** Esra İpekci, Emre Ş. Çağlar, Mustafa S. Kaynak, Evren Gündoğdu, Neslihan Üstündağ Okur

**Affiliations:** † Department of Pharmaceutical Technology, Faculty of Pharmacy, 448249 University of Health Sciences, İstanbul 34668, Turkey; ‡ Department of Pharmaceutical Biotechnology, Faculty of Pharmacy, University of Health Sciences, İstanbul 34668, Turkey; § Department of Pharmaceutical Technology, Faculty of Pharmacy, Anadolu University, Eskişehir 26470, Turkey; ∥ Department of Radiopharmacy, Faculty of Pharmacy, Ege University, İzmir 35040, Turkey

## Abstract

The purpose of this study was to develop and evaluate novel nadolol (NDL)-loaded microemulsions for oral application. Microemulsions were prepared using a pseudoternary phase diagram through the aqueous titration method. The optimum formulation constituted a mixture of cinnamon oil as the oil phase, Tween 20 as the surfactant, propylene glycol:ethanol as cosurfactants, and distilled water as the water phase. The selected formulation was loaded with 4% NDL. Droplet size, polydispersity index (PDI), ζ-potential, conductivity, pH, refractive index, viscosity, thermodynamic stability, storage stability, and *in vitro* drug release were evaluated in characterization studies. Cell culture and *in situ* single-pass intestinal perfusion (SPIP) were investigated. Cell studies were performed in order to understand the cytocompatibility by using the (3-[4,5-dimethylthiazol-2-yl]-2,5-diphenyltetrazolium bromide) method and the permeation of NDL by using the Caco-2 cell line. To estimate the permeability values, SPIP was carried out with female Sprague–Dawley rats. An ideal NDL-loaded microemulsion formulation showed an average 1.01 ± 0.07 nm droplet size, 0.46 ± 0.04 PDI, and −10.26 ± 0.87 mV ζ-potential. pH, viscosity, conductivity, and refractive index values of an ideal NDL-loaded formulation were found to be 5.88 ± 0.02, 76.67 ± 5.77 cP, 201.70 ± 0.00 μS/cm, and 1.42 ± 0.00, respectively. In the SPIP study, an ideal NDL-loaded microemulsion formulation was found to have 5.826 times higher NDL permeability compared to a pure NDL solution, and the estimated fraction of the substance absorbed in humans was determined to be 39.821 ± 9.202%. It was found to have suitable thermodynamic and storage stability. This study showed that NDL-loaded microemulsions can serve as promising oral drug delivery systems for antihypertensive purposes. The developed NDL-loaded microemulsion significantly improved permeability and predicted oral absorption while maintaining safety, indicating strong potential as an effective oral formulation. These results suggest that it may enhance therapeutic outcomes in hypertension, although further *in vivo* and clinical studies are needed.

## Graphical abstract

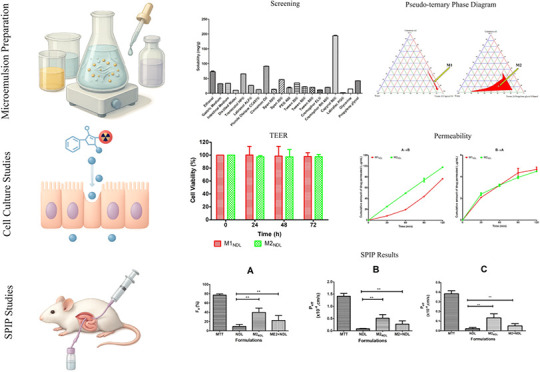

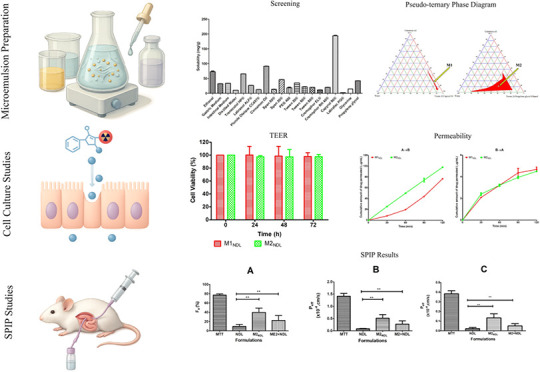

## 1 Introduction

Hypertension is the leading modifiable risk factor for global morbidity and mortality, accounting for about 29% of cardiovascular deaths, and is expected to affect nearly 33% of the population by 2025 due to increasing obesity and aging.^1–3^ It is defined as a systolic blood pressure higher than 140 mmHg or a diastolic blood pressure higher than 90 mmHg, and occurs either as secondary hypertension or, more commonly (90%), as essential hypertension.^4,5^ As a chronic condition, it requires long-term pharmacological treatment.

Using oral drugs is a comfortable way to treat diseases that require long-term treatment, such as hypertension. In addition, the oral route is a more preferred method of drug administration because of its patient-friendliness, safety, cost-effectiveness, flexibility in dosage adjustment, and suitability for long-term use.^6–8^ Oral therapy for hypertension commonly employs representatives of multiple pharmacologic categories: thiazide-based diuretics, β-adrenoceptor antagonists, angiotensin-converting enzyme inhibitors, angiotensin II receptor antagonists, and L-type calcium-channel antagonists.^9^ According to the ESC/ESH guidelines, β-blockers are a group included in the first-line treatment in hypertension patients with certain indications.^10^

β-adrenergic receptor antagonists, commonly known as β-blockers, are widely prescribed for hypertension, arrhythmias, and coronary heart disease. Nadolol (NDL), (2*R*,3*S*)-5-[3-(*tert*-butylamino)-2-hydroxypropoxy]-1,2,3,4-tetrahydronaphthalene-2,3-diol, is an active ingredient in the group of nonselective β-blockers.^11^ In addition to the treatment of hypertension, it is used in the clinic to prevent angina pectoris^12^ and esophageal varices, to treat hereditary or familial essential tremor disease, and to reduce peripheral physiological symptoms of anxiety^13^ and migraine prophylaxis.^14^ NDL is present in conventional tablet preparation used orally in the market. Its bioavailability is around 30%.^15^ In oral drug design, exposure to different conditions of the gastrointestinal tract such as pH change from acidic to basic, various enzymes and surfactants (such as lipases, pepsinogens, pepsins, bile, etc.), gastrointestinal microbiota, mucus barrier, and epithelial layer results in low bioavailability.^8,16^ Nadolol (NDL) is classified as a Biopharmaceutics Classification System (BCS) Class III drug, characterized by high solubility but low intestinal permeability. This limited permeability represents a major barrier to oral bioavailability. The lipophilicity of NDL, expressed as its partition coefficient (log P), has been reported to be relatively low, supporting its poor membrane permeability behavior. Therefore, formulation strategies that can enhance intestinal permeability are of particular interest.^12,17^ In order to overcome these challenging conditions, there is a need for a new generation of oral drug delivery systems such as microcapsules, microspheres, microsponges, nanoparticles, polymeric micelles, dendrimers, carbon nanotubes, solid lipid nanoparticles, liposomes, and microemulsions^18^ with a lower side effect profile and more effectiveness.^19^ While studies specifically exploring microemulsion-based formulations of nadolol are limited, evidence from other drugs suggests that microemulsion systems can improve intestinal uptake and pharmacokinetic profiles by enhancing membrane transport and lymphatic uptake.^20,21^

Microemulsions are single-phase (isotropic) and transparent systems composed of oil, water, and surfactant phases. The surfactant reduces the surface tension of these two immiscible phases and allow them to disperse among each other.^22,23^ These systems form spontaneously with mild shaking, have a droplet size of 10–200 nm,^24^ and are thermodynamically stable.^25,26^ The small droplet size increases the bioavailability of the active substance and helps absorb faster in the gastrointestinal tract without going through disintegration stages like tablets.^27^ Microemulsions are easy to prepare,^26,28^ have a Newtonian flow,^24^ protect the molecules against enzymatic oxidation and hydrolysis,^19^ increase the solubility of the active ingredient, block molecules associated with P glycoprotein (multidrug-resistant proteins) in many ways, and increase drug absorption.^19,29^ They are a suitable carrier system^19^ for both lipophilic and hydrophilic active ingredients, and these systems provide ease of scale-up.^29^ Their unique properties, including low interfacial tension and the ability to solubilize hydrophobic and hydrophilic components, make them versatile candidates for oral drug delivery. Microemulsion-based formulations have been shown to enhance gastrointestinal absorption and bioavailability, particularly for drugs with limited intestinal permeability.^20,30^

Plant-derived oils, such as cinnamon oil (*Cinnamomum* spp.), have gained attention as the oil phase in microemulsions due to their bioactive constituents, including terpenes and phenolic compounds. Beyond serving as a carrier, cinnamon oil may provide antioxidant and other biological effects.^31^ Cinnamon oil-based microemulsions have been evaluated for stability, antimicrobial activity, and antioxidant properties, demonstrating that the oil phase can support both drug delivery and potential therapeutic benefits.^31,32^ Although studies specifically assessing cinnamon oil microemulsions for antihypertensive applications are limited, their incorporation into oral nadolol formulations could offer an innovative strategy to improve the intestinal absorption and overall pharmacological performance.

Overall, developing oral microemulsion systems for hydrophilic β-blockers, including nadolol, particularly those incorporating bioactive oils such as cinnamon oil, represents a promising and underexplored approach. These systems have the potential to enhance intestinal permeability, optimize pharmacokinetics, and introduce additional therapeutic benefits, which highlight their relevance in contemporary pharmaceutical research. This work comprises the development, evaluation, and characterization of NDL-loaded microemulsions. Moreover, the developed formulations were supported with *in vitro* release, cell culture permeability, and cytocompatibility and SPIP studies. These drug delivery systems aim to increase the bioavailability of NDLs and serve as an alternative to tablet forms on the market.

## 2 Materials and Methods

### 2.1 Materials

Cinnamon oil, Tween 20 (polysorbate 20), ingredients of Golytely solution NaCl (sodium chloride), KCl (potassium chloride), Na_2_SO_4_ (sodium sulfate), NaHCO_3_ (sodium hydrogen carbonate), mannitol, orthophosphoric acid, and methanol were purchased from Sigma (Germany). Propylene glycol, n-octanol, hydrochloric acid, phenol red (PR), and metoprolol tartrate (MPT) were purchased from Merck (Germany). NDL was purchased from ChemCruz (USA). Capryol 90 (propylene glycol monocaprylate) was gifted from Gattefosse (France). Sodium chloride was from ISOLAB Chemicals (Germany). Dulbecco’s modified Eagle medium and Hank’s balanced salt solution (HBSS) were purchased from Gibco (England). Analytical-grade standards were met for every reagent and each buffer component, and distilled water was employed across all experimental procedures.

### 2.2 Solubility of Excipients

A screening of NDL solubility at 25 ± 2 °C was undertaken to nominate suitable oil–surfactant mixtures for microemulsions, encompassing ethanol, gastric medium, intestinal medium, distilled water, Transcutol HP (2-(2-ethoxyethoxy)­ethanol), Labrasol ALF (caprylocaproyl macrogol-8 glycerides), Plurole Oleique CC497 (polyglyceryl-3 dioleate), cinnamon oil, Span 80 (sorbitan oleate 80), Span 20 (sorbitan oleate 20), PEG 400 (polyethylene glycol 400), Tween 20, Tween 60, Tween 80, Cremophor EL (polyoxyethylene glycerol triricinoleate), Cremophor RH 40 (PEG-40 hydrogenated castor oil), Capyrol 90, Labrafac PG (propylene glycol dicaprolate), glycerin, and propylene glycol. To prepare saturated solutions, an excess of NDL was distributed among the solvents (500 mg each) and agitated for 24 h at a controlled 25 ± 2 °C, followed by centrifugation for 30 min at 15000 rpm (Sigma 3-18 KS). The resultant supernatant, after dilution with the mobile phase, was assayed for drug content via high-performance liquid chromatography (HPLC). Excipients yielding greater NDL solubilization were prioritized as microemulsion components.^33^

### 2.3 Determination of the *n*-Octanol/Water Partition Coefficient

For assessment of the partition coefficient (*n*-octanol/water), 10 mg of NDL was placed in 32 mL of *n*-octanol–water mixture; samples were shaken for 24 h at ambient temperature and then centrifuged at 3500 rpm for 15 min. The drug concentration was quantified by HPLC, and all determinations were carried out in triplicate.

### 2.4 HPLC Analysis

An Agilent 1200 configuration (HPLC platform) incorporating an isocratic pump, a temperature-controlled column compartment, and a UV detector was employed. Chromatography utilized a C18 column (5 μm, 250 × 4.6 mm^2^; Phenomenex). For characterization and *in vitro* drug-release experiments, analyses were performed at 206 nm with a flow rate of 1 mL/min at 25 ± 2 °C. The injection volume was 20 μL. The mobile phase consisted of 0.293% NaCl:methanol (65:35) to which 1 mL of HCl was added. The retention time of the drug was 5.6 min.^34^

### 2.5 Preparation of the Pseudoternary Phase Diagram and Microemulsions

To establish composition optima consistent with the solubility results, pseudoternary phase plots were obtained via a titration protocol. Visual inspection was performed as distilled water was added to an oil with a surfactant/cosurfactant mixture at ambient temperature. Surfactant:cosurfactant (w/w) ratios were 1:1, 1:2, 1:3, 1:4, 1:5, 1:6, 1:7, 1:8, and 1:9. Pseudoternary phase diagrams were drawn with software (Triangle phase diagram analysis software).^35^ The resulting systems were visually evaluated to determine the formation of the microemulsions. On the basis of the microemulsion region in the phase diagrams, appropriate formulations were selected and developed.^36,37^

### 2.6 Preparation of NDL-Loaded Microemulsions

Optimum microemulsions were selected according to the data obtained by the solubility and phase diagram studies. The oil, surfactant, and cosurfactant were mixed in the specified proportions, and distilled water was added dropwise and shaken gently. The formulations contained 4% (w/w) of NDL.

### 2.7 Characterization of Microemulsions

Characterization of the formulations encompassed measuring the droplet size, polydispersity index (PDI), ζ-potential, electrical conductivity, pH, refractive index, viscosity, *in vitro* release, and thermodynamic stability.

#### 2.7.1 Droplet Size, PDI, ζ-Potential, and Electrical Conductivity Analysis

Droplet size, PDI, ζ-potential, and electrical conductivity were measured by a Malvern Zetasizer Ultra (Malvern Instruments, U.K.) in backscatter mode. Droplet size and PDI reflect the mean of 10 runs acquired at 173° detection angle in PCS8501 disposable cells with the temperature held at 25 °C. For ζ-potential, both the blank and drug-loaded microemulsions were analyzed in DTS1070 ζ-cells on the same platform with the dielectric constant set to 78.5 and the temperature maintained at 25 °C. Each sample underwent three replicate measurements; reported values are the means.^38^

#### 2.7.2 Determination of pH

A Mettler Toledo (Switzerland) pH meter was employed to assess the pH of each formulation at 25 ± 2 °C. Three measurements per sample were obtained, and averages were recorded.^39^

#### 2.7.3 Determination of the Refractive Index

A refractometer (A. Krüss Optronic DR301-95) was used to quantify the samples’ refractive index. Each assay comprised three replicates conducted at 25 ± 2 °C, and the arithmetic mean was taken.^39^

#### 2.7.4 Determination of Viscosity

A Brookfield Ametek DVE viscosimeter was employed to assess sample viscosity. Each formulation was analyzed at 25 ± 2 °C with spindle no. 06 (100 rpm), and measurements were obtained three times per sample.^40^

#### 2.7.5 Thermodynamic Stability Studies

For physical stability, samples were tested by centrifugation (Sigma 3-18 KS). The formulations were subjected to centrifugation at 4100 rpm for 30 min. Experiments were performed in triplicate.^41^

The samples were kept at 4 ± 2 °C for 24 h under cooling and at 40 ± 2 °C for 24 h under heating. The formulations were subjected to cooling (4 °C, 24 h) and heating (45 °C, 24 h) cycles three times and then centrifuged for 5 min at 3000 rpm. The samples were investigated in terms of phase separation, turbidity, and precipitation. Experiments were performed in triplicate.^42^

The samples were left to freeze at −20 ± 2 °C for 24 h and then stored at 40 ± 2 °C for 24 h. After centrifugation at 3000 rpm, the samples were evaluated in terms of phase separation, turbidity, and precipitation. Experiments were performed in triplicate.^42^

### 2.8 *In Vitro* Drug Release Studies

The dialysis bag (Spectra/Por Dialysis membrane, 12–14 kDa MWCO) method was used in the *in vitro* drug release study. Gastric (pH 1.2) and intestinal media (pH 6.8) were used as release medium. Microemulsion formulations containing 40 mg/mL NDL were prepared, providing sink conditions at 37 ± 0.5 °C and capped with closures. The membrane was kept on hold in saline before use. 1 mL of microemulsion formulations was added to the dialysis bag and immersed in 99 mL of gastric and intestinal media containing 20% ethanol (v/v) to maintain sink conditions. This system was mixed with a magnetic stirrer at 150 rpm. 500 μL samples were taken at predetermined times (in the gastric media at 0.25, 0.5, 0.75, 1, 2, 3, 4, 6, and 8 h and in the intestinal media at 0.25, 0.5, 0.75, 1, 2, 3, 4, 6, 8, 10, 12, and 24 h) and analyzed by HPLC. The system was completed with gastric and intestinal media at the same amount and temperature in each sample collection. Experiments were performed in triplicate.^42^

To assess the *in vitro* drug release kinetics of the various formulations, the model-independent mathematical framework proposed by Moore and Flanner was utilized. This technique relies on two primary metrics: the difference factor (*f*_1_) and the similarity factor (*f*_2_) to distinguish between the dissolution behaviors. According to established criteria, the release patterns are considered similar when *f*_1_ results remain under 15 and *f*_2_ values fall within the 50–100 range.^43^ Additionally, the study involved statistical evaluations to determine the intraformulation consistency.

### 2.9 Permeability Studies in the Caco-2 Cell Monolayer

DMEM-maintained Caco-2 cultures, spanning passages 21 through 55, were used to establish monolayers by seeding 1 × 10^5^ cells onto Transwell inserts in six-well plates; environmental conditions were 37 °C, 90% humidity, and 5% CO_2_, with experiments initiated 21 days after plating. Monolayer integrity was confirmed by transepithelial electrical resistance (TEER) measurement employing an epithelial voltameter (EVOM, World Precision Instrument, Sarasota, FL) before and after each assay.

Formulation permeability was then examined under identical atmospheric conditions with medium changes every other day for 3 weeks, using bidirectional protocols (A–B and B–A). Following two washes with prewarmed HBSS (pH 7.4), the apical compartment (A, 2.2 mL) was dosed for A–B studies and the basolateral compartment (B, 3.2 mL) was dosed for B–A studies. Samples were obtained from the receiver compartment after 2 h at 37 °C with orbital mixing at 50 rpm in a water bath and analyzed by HPLC. For each formulation, three parallel release studies were performed.^44^

### 2.10 SPIP Studies

#### 2.10.1 Intestinal Perfusion Experimental Procedure

Experiments involving animals were performed in accordance with protocols sanctioned by the Anadolu University Committee of Use and Care of Animals (Protocol no. 2021-43). The surgical procedures and experiments were similar to Okur et al.^45^ Perfusion experiments were performed using female rats of the Sprague–Dawley strain (250–350 g; *n* = 30). Before the experiment, free-access water, fasted rats were kept under a 12 h light–dark cycle. The body temperature of the animals was kept constant at 37 °C before and throughout the experiment. Surgical procedures were performed under anesthesia. Anesthesia was provided by intraperitoneal administration of a ketamine hydrochloride (90 mg/kg)–xylazine hydrochloride (5 m/kg) combination. Under anesthesia, the abdominal membrane was cut, the intestines were exposed, and the ileum was detected. The segment length to be perfused was set as 9.0–10.0 cm for the ileum. Silicone cannulae were installed on the two extremities of the intestinal segment after isolation, one proximal and the other distal, and tied in place with a suture material. The isolated segment was cleaned by washing with a perfusion solution (37 °C) and a clear solution was taken as an indicator of successful cleaning. The flow rate was set to be 0.2 mL/min via the peristaltic pump, and the perfusion solution was not circulated during the experiment.^46–49^

#### 2.10.2 Perfusion Solution

Golytely, iso-osmotic solution, was used as perfusion medium in all perfusion studies. Golytely content is given in Table 1. The perfusion solution was prepared fresh, adjusted to pH 7.4 with orthophosphoric acid, and used after filtering through a membrane filter with a diameter of 0.45 μm.^47^

**Table 1:** Composition of the Perfusion Solution Used in Intestinal Perfusion Studies

content	concentration (mmol/L)
NaCl	25
KCl	10
Na_2_SO_4_	40
NaHCO_3_	20
mannitol	80

In experiments, 160 ppm NDL, 100 ppm PR, and 400 ppm MPT was used as the model drug, zero permeability agent, and reference drug, respectively. The solutions were prepared weekly and freshly and kept in the fridge at 4 °C.^49^

#### 2.10.3 Perfusion Experiments

The SPIP procedure was applied. Perfusion studies were performed on three groups. The concentrations of NDL (model drug), MT (reference drug), and PR (zero permeability agent) were determined by HPLC.

In group 1, NDL was added to the perfusion solution, and the ileum was perfused for 60 min. Samples were collected every 10 min, and the amounts of MTP, PR, and NDL in the samples were determined by an improved HPLC method. With this group, the permeability class of the active substance according to the BCS was determined.

In group 2, the M2_NDL_ formulation was added to the perfusion solution and the ileum was perfused for 60 min, and the amount of NDL was determined by an improved HPLC method.

In group 3, NDL and the blank M2 formulation were added to the perfusion solution, the ileum was perfused for 60 min, and the amount of NDL was determined by an improved HPLC method.

#### 2.10.4 HPLC of SPIP Samples

For SPIP studies, the modified method and a different HPLC device were used. The HPLC system consisted of an isocratic pump, temperature adjustable column, and UV detector supplied by Shimadzu. A Phenomenex C18 column (5 μm, 250 × 4.6 mm^2^) was used. The samples were analyzed at 206 nm with 1.6 mL/min at 25 ± 2 °C flow rate. The injection volume of the samples was 50 μL. The mobile phase was prepared by adding 1 mL of HCl in a mixture of 0.293% NaCl:acetonitrile (78:22). Both analytical methods underwent partial validation encompassing evaluations of linearity; limits of detection and quantitation (LOD, LOQ); precision; accuracy; specificity/selectivity; and stability.

#### 2.10.5 Data Analysis

To evaluate differences between the two experimental cohorts, a nonparametric, two-sided Mann–Whitney *U* procedure was applied, adopting *p* < 0.05 as the significance threshold.

The experimental results obtained were calculated with the following equations.^45,49^

- Effective permeabilities (*P*_eff_) of NDL and MPT were calculated using eq 1
1 $$P_{\mathrm{eff}}=\frac{-Q\ln{\left[\frac{C_{\mathrm{out}}}{C_{\mathrm{in}}}\right]}^{\prime }}{2\pi \mathrm{rl}}$$
*Q*: flow rate (mL/s),
  (*C*_out_/*C*_in_)^l^: outlet concentration and the inlet (reservoir) concentration of NDL (corrected for the water transport),
*l*: perfused segment (cm), and
*r*: radius of the perfused segment (cm) (0.18 cm for the ileum).
- *C*_out_/*C*_in_ of NDL was calculated using eq 2
2 $$\left[\frac{C_{\mathrm{out}}}{C_{\mathrm{in}}}\right]=\left[\left(\frac{C_{\mathrm{out}}}{C_{\mathrm{in}}}\right)\mathrm{drug}.\left(\frac{C_{\mathrm{out}}}{C_{\mathrm{in}}}\right)\mathrm{PR}\right]$$.

$\left(\frac{C_{\mathrm{out}}}{C_{\mathrm{in}}}\right)\mathrm{PR}$ : the ratio of concentration of PR in the inlet sample and concentration of PR in the outlet sample.
- The net water flux (NWF) values were calculated using eq 3
3 $$\mathrm{NWF}=\frac{\left[1-\left(\frac{C_{\mathrm{out PR}}}{C_{\mathrm{in PR}}}\right)\right].Q_{\mathrm{in}}}{l}$$
- The human *P*_eff_ value was calculated using eq 4
4 $$P_{\mathrm{eff}\;\mathrm{humans}}=3.6P_{\mathrm{eff}\;\mathrm{rats}}+0.03\times 10^{-4}$$
- The fraction absorbed (F_a_) in humans was calculated using eq 5
5 $$F_{a}=1-e^{-3845.P_{\mathrm{eff rats}}}$$

### 2.11 Storage Stability Studies

The storage stability studies were carried out at 5 ± 3 °C, 25 ± 2 °C/60 ± 5% relative humidity (RH), and 40 ± 2 °C/75 ± 5% RH for 12 months. Physical appearance, droplet size, PDI, ζ-potential, and drug content were measured at 0, 1, 3, 6, and 12 months.^35,41^

### 2.12 Statistical Analysis

Statistical analyses were performed to evaluate the significance of the results. The paired *t* test was used for dependent pairs, while one-way ANOVA followed by Tukey’s posthoc test was applied for comparisons involving more than two groups. For nonparametric data involving two independent groups, the Mann–Whitney *U* test was utilized. Results were considered significant at *p* < 0.05.

## 3 Results and Discussion

### 3.1 Solubility Studies

Solubility studies were carried out to detect the components with which NDL is highly soluble, contributing to increased oral bioavailability.^50^ The best solubility of NDL appeared in Capryol 90 (193.93 ± 1.53 mg/g). Cinnamon oil (91.4 ± 0.34 mg/g) as the oil phase, Tween 20 (34.86 ± 0.02 mg/g) as the surfactant, and Capryol 90 (193.93 ± 1.53 mg/g), propylene glycol (42.17 ± 0.18 mg/g), and ethanol (73.09 ± 2.35 mg/g) as cosurfactants are chosen as microemulsion components. The results of the solubility study are given in Figure 1.

**Figure 1:**
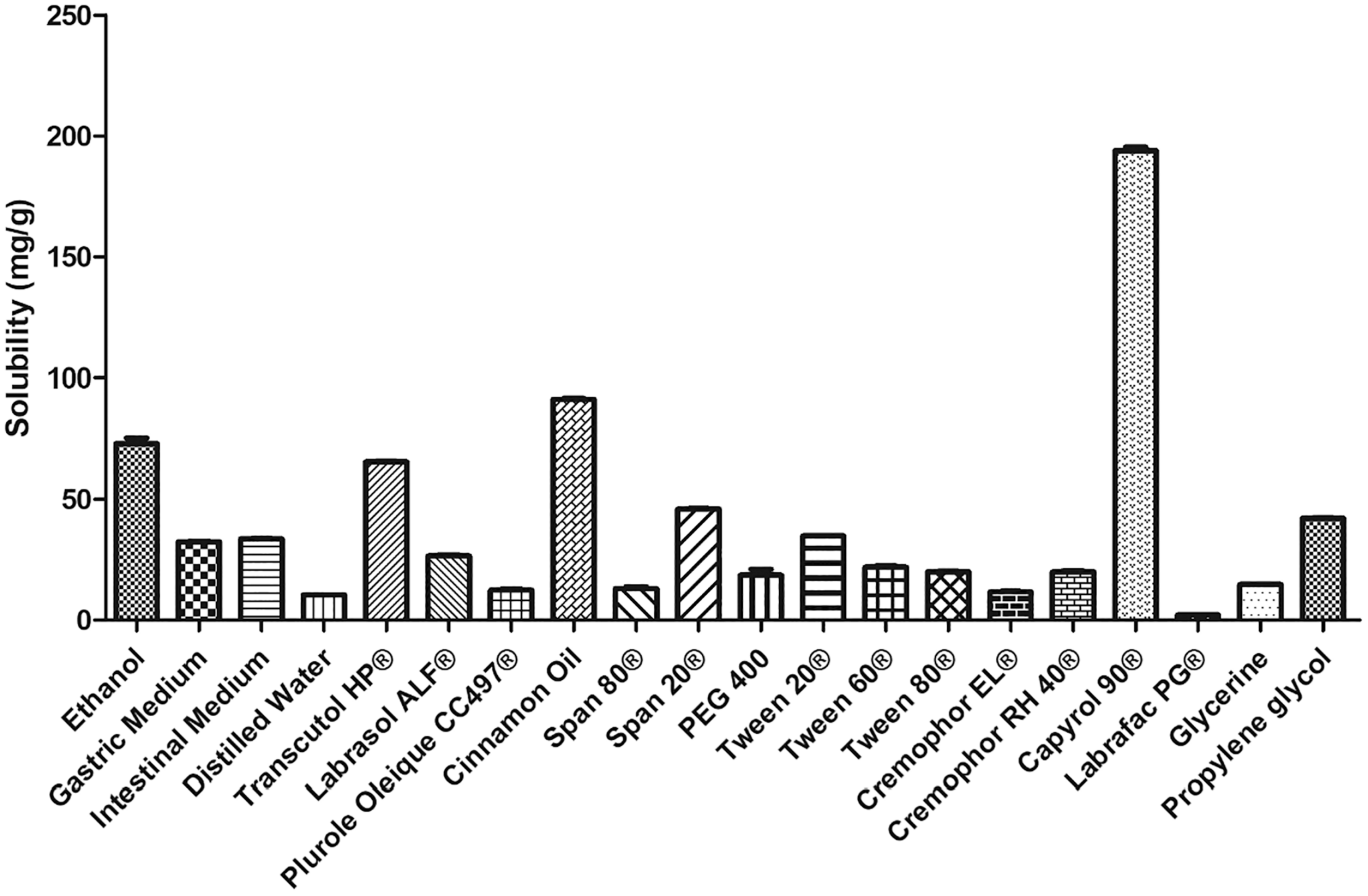
Solubility results of NDL in different components and media (±SD).

The ingredients used in the formulation design are essential as they directly affect the solubility, bioavailability, and toxicity of the formulation and the active substance. The oil obtained from the leaves of *Cinnamomum zeylanicum* was used in the study. Cinnamon oil, which contains cinnamic acid, eugenol, cinnamaldehyde, and cineole active molecules, has anticancer, antidiabetic, anti-inflammatory, antibacterial, and antifungal effects. Additively, cinnamaldehyde and eugenols show beneficial effects in cell destruction, heart disease, and hypertension disease through antioxidant effects.^51,52^

Tween 20 (HLB 16.7) is a polysorbate class excipient. Polysorbates have been reported as safe, biocompatible, nonionic excipients and convenient for microemulsion formulations.^52–54^

Cosurfactants provide adequate interfacial flexibility and improve surfactant emulsification.^55,56^ Propylene glycol is one of the used excipients in the pharmaceutical industry with a solvent because of the extractant and preservative properties.^52^ The use of nontoxic doses of ethanol increases drug permeability with the drug passing through by improving membrane permeability in oral drug delivery systems.^57^ Propylene glycol and ethanol are commonly used in oral drug delivery systems.^58^ Capryol 90, which is used as another cosurfactant, increases the bioavailability by opening tight junctions.^59^

### 3.2 Determination of the *n*-Octanol/Water Partition Coefficient

The calculated log P value of NDL was found to be 1.283 ± 0.193. The obtained log *P* value supports that NDL is a low-permeability compound in the BCS class III group.^17^

### 3.3 HPLC Analysis

The HPLC method was developed and validated for NDL determination. It was studied in the calibration range of 1–100 μg/mL (ppm) in HPLC. The *R* ^2^ value for the regression line is 0.9996, and the standard line equation of NDL, in the mobile-phase environment, is *y* = 68.525*x* + 88.552. Since the coefficient of variation for precision, accuracy, and reproducibility is below 2%, the analytical method was deemed appropriate. Moreover, the method meets the criteria of specificity and durability. LOD and LOQ values were determined as 0.452 μg/mL and 1.396 μg/mL, respectively.

### 3.4 Preparation of the Pseudoternary Phase Diagram and Microemulsions

On the basis of the solubility study, cinnamon oil as the oil phase, Tween 20 as surfactants, Capryol 90, propylene glycol, and ethanol as cosurfactants, and distilled water as the aqueous phase were selected. The ratios of the selected components were optimized by drawing a pseudoternary phase diagram. A pseudoternary phase diagram was constructed via an aqueous titration method to select a suitable ratio of surfactant:cosurfactant. Blank microemulsions, which showed a larger area than that of the others in the pseudoternary phase diagram, were selected and are shown in Figure 2.

**Figure 2:**
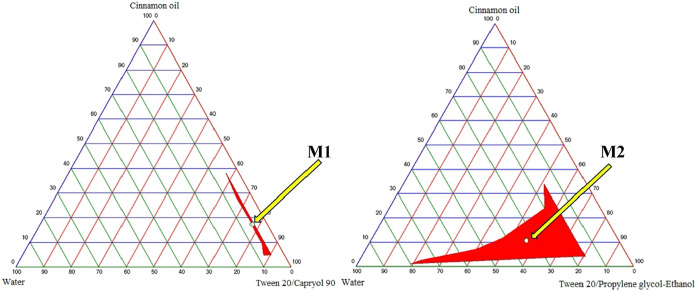
Pseudoternary phase diagram of the microemulsion formulations (the white point shows the optimum formulation ratio).

M1 and M2 show 28.29 and 495.9 area values, respectively (Figure 2). The region’s center of gravity in the pseudophase diagram represents optimum component ratios. The hydrophilic–lipophilic balance (HLB) value used in the classification of surfactants and as an indicator of the emulsion type varies between 0 and 20.^60^ At high HLB values, the formulation is prone to the formation of oil-in-water microemulsions. In oral administration, due to the aqueous nature of the gastrointestinal environment, formulations with a high HLB value provide better dispersion and stability.^61^ HLB values and optimum ratios are given in Table 2.

**Table 2:** Compositions and HLB Values of Microemulsion Formulations

component	M1 (%) (w/w)	M2 (%) (w/w)
cinnamon oil	17.22	10.65
Tween 20	15.48	28.05
Capryol 90	61.90	
propylene glycol:ethanol (3:1)		28.05
distilled water	5.40	33.25
HLB	8.14	12.66

### 3.5 Characterization of Microemulsions

The appearance of the freshly prepared microemulsions with and without NDL formulations was observed visually. M1, M1_NDL_, M2, and M2_NDL_ appeared clear, transparent, and homogeneous. The color of the formulations is yellowish due to the cinnamon oil.

A small and homogeneous globule size is among the important parameters for the stability of the microemulsion.^62^ The average droplet sizes of M1, M2, M1_NDL_, and M2_NDL_ were 197.10 ± 9.52 nm, 8.01 ± 0.12 nm, 97.04 ± 3.31 nm, and 1.01 ± 0.07 nm, respectively (Table 3). NDL significantly decreased the droplet size of formulations. The PDI is the heterogeneity degree of the formulation droplet size. The PDI of M1, M2, M1_NDL_, and M2_NDL_ was 0.30 ± 0.05, 0.28 ± 0.18, 0.43 ± 0.27, and 0.46 ± 0.04 respectively (Table 3). One of the factors affecting the droplet size is increasing oil concentration. This factor supports the smaller droplet size of the M2 formulation.^63^ Since the PDI is less than 0.5, it can be said that the droplet sizes of the formulations have acceptable homogeneity.^64^

**Table 3:** Droplet size, PDI, ζ-Potential, Electrical Conductivity, pH, Viscosity, and Refractive Index of Microemulsion Formulations (±SD)

formulation	M1	M1_NDL_	M2	M2_NDL_
droplet size (nm)	197.10 ± 9.52	97.04 ± 3.31	8.01 ± 0.12	1.00 ± 0.07
PDI	0.30 ± 0.05	0.43 ± 0.27	0.28 ± 0.18	0.46 ± 0.04
ζ-potential (mV)	0.08 ± 0.01	–3.15 ± 2.68	0.73 ± 0.07	–10.26 ± 0.87
conductivity (μS/cm)	2.21 ± 0.00	7.81 ± 0.00	74.70 ± 0.00	201.70 ± 0.00
pH	6.52 ± 0.02	5.72 ± 0.01	6.48 ± 0.01	5.88 ± 0.02
viscosity (cP)	72.00 ± 4.47	66.67 ± 5.77	98.00 ± 4.47	76.67 ± 5.77
refractive index	1.45 ± 0.01	1.45 ± 0.00	1.42 ± 0.01	1.42 ± 0.00

Franklyne et al. defined a microemulsion as a uniform, transparent, thermodynamically stable system with a droplet size less than 100 nm. The droplet size of the microemulsion formulations developed by Franklyne et al. was between 10 and 19.3 nm, and the PDI was analyzed below 0.4.^65^ The microemulsion developed by Suthar et al. is monophasic and transparent and has a droplet size of about 14 nm and a low PDI value. It has been reported that these formulations can easily pass through biological membranes because of their small droplet size.^66^ Small droplet size makes the microemulsion more stable against conditions such as creaming and phase separation. Koli et al. reported that as the oil phase ratio increased, the droplet size increased. In this study, M2 has less oil phase and a smaller droplet size.^7^

Droplet size measurements were performed using a Zetasizer based on dynamic light scattering (DLS), which provides the hydrodynamic diameter of dispersed structures. The droplet size obtained for M2_NDL_ was lower than the typical range reported for microemulsions. It is known that DLS measurements may also reflect very small colloidal structures, particularly in systems containing surfactants and cosolvents. Therefore, the observed value can be associated with the specific composition of the formulation, as well as the measurement technique. Similar considerations have been reported in the literature.^67^

ζ-potential is an indicator of the degree of electrostatic repulsion between droplets or particles in the dispersion medium and the stability of the colloidal system. Increasing ζ-potential increases the repulsion between droplets/particles and prevents aggregation tendency.^51,59^ The ζ-potential of M1, M1_NDL_, M2, and M2_NDL_ was analyzed as 0.08 ± 0.01 mV, −3.15 ± 2.68 mV, 0.73 ± 0.07 mV, and −10.26 ± 0.87 mV, respectively (Table 3). The ζ values of the formulations were measured close to neutral. In many studies in the literature, it has been mentioned that the neutral ζ-potential is due to the nonionic components used.^68^

The ζ-potential of piroxicam-loaded microemulsions developed by Xing et al. was found to be between −6.41 mV and −7.76 mV.^69^ Patel et al. analyzed the ζ-potential of clopidogrel-loaded microemulsions as −6.34 mV. It was stated that aggregation is not expected in the microemulsion due to the slightly negative ζ-potential.^70^

Electrical conductivity is one of the parameters used to determine the microemulsion type. The M2 formulation is an oil-in-water microemulsion due to its higher water phase ratio and conductivity over 52.5 μS/cm.^71^ The electrical conductivity is directly related to the water ratio of the formulation The measured electrical conductivities are given in Table 3. The active substance loading increased the electrical conductivity. As can be seen, the high water content in M2 caused high electrical conductivity.^22^ You et al. studied ibuprofen-loaded microemulsions. Similar to the study, the high conductivity value attributed to the formulations being oil-in-water emulsions was found to be over 100 μS/cm.^72^

The refractive index values of M1, M1_NDL_, M2, and M2_NDL_ were measured as 1.45 ± 0.01, 1.45 ± 0.00, 1.42 ± 0.01, and 1.42 ± 0.00, respectively (Table 3). There is no remarkable difference between M1 and M2 values. NDL loading of formulations caused almost no change in the refractive index. Alam et al. drew attention to the effects of the water content of formulations on conductivity and refractive index. It has been reported that the conductivity increases with increasing water ratio in the microemulsions. Similarly, in the study, the M2 formulation had a higher water content and showed higher conductivity than that of M1. In addition, it was reported that the refractive index of formulations with higher water content showed values close to that of deionized water (1.33). Similarly, the refractive index of the M2 formulation, which has a higher water content, was found to be closer to deionized water.^73^

The viscosities of M1 and M2 are 72.00 ± 4.47 and 98.00 ± 4.47, respectively (Table 3). NDL loading in the formulations decreased the viscosity. The viscosity of M1_NDL_ and M2_NDL_ was measured as 66.67 ± 5.77 cP and 76.67 ± 5.77 cP, respectively (Table 3). Shah et al. loaded microemulsions to improve the oral bioavailability of raloxifene. Viscosity in microemulsions formed by using Tween 20 as a surfactant was measured at close values (83 ± 12 cP) in this study. Shah et al. stated that this viscosity value provides comfort in transportation and packaging, which is essential in drug design for oral use.^74^

The pH values of M1 and M2 formulations were measured as 6.52 ± 0.02 and 6.48 ± 0.01, respectively. NDL loading slightly decreased the pH, resulting in the following: M1_NDL_: 5.72 ± 0.01 and M2_NDL_: 5.88 ± 0.02. Koli et al. developed felodipine-loaded microemulsion. Tween 20 was used as a surfactant in the study. Similarly, it was determined that the pH values of the loaded formulations varied between 6.02 and 6.59 and these values were reported to be suitable for oral use.^7^

The droplet size, PDI, ζ-potential, electrical conductivity, pH, viscosity, and refractive index value results of the formulations are given in Table 3.

#### 3.5.1 Thermodynamic Stability Studies

Centrifugation, heating (4 ± 2 °C)–cooling (40 ± 2 °C) cycles, and freezing (−20 ± 2 °C)–thawing (40 ± 2 °C) cycles were carried out in thermodynamic stability studies. Durgapal et al. reported that the thermodynamic stability of microemulsions is affected by the well-optimized microemulsion components, especially the Smix ratio.^75^ As a result of these studies, no physical stability problem such as phase separation was observed except for M1. The M2 formulation was considered thermodynamically stable.

### 3.6 *In Vitro* Drug Release Study

An *in vitro* release assay is a useful method in obtaining information such as the release mechanism and kinetics of nanocarriers.^76^ An *in vitro* drug release study was evaluated in gastric (pH 1.2) and intestinal media (pH 6.8) using a dialysis membrane. Release results were analyzed by using HPLC.

At 4 h, which is the gastric emptying time, the formulations released 100% of their content. M1_NDL_ was released 100% in 1 h, while M2_NDL_ was released 100% in 3 h. The M2_NDL_ formulation was released more slowly. In the intestinal environment, both formulations reached 100% release rate in the third hour. The formulations showed very close release rates. The formulations showed a faster release rate in the gastric environment. Curcumin-loaded microemulsions were developed and studied for *in vitro* drug release by Wang et al. Similarly, the formulations showed a higher release rate in the gastric environment. It has been stated that this is due to the deterioration of the microemulsion structure at acidic pH.^77^ The results obtained are listed in Figure 3.

**Figure 3:**
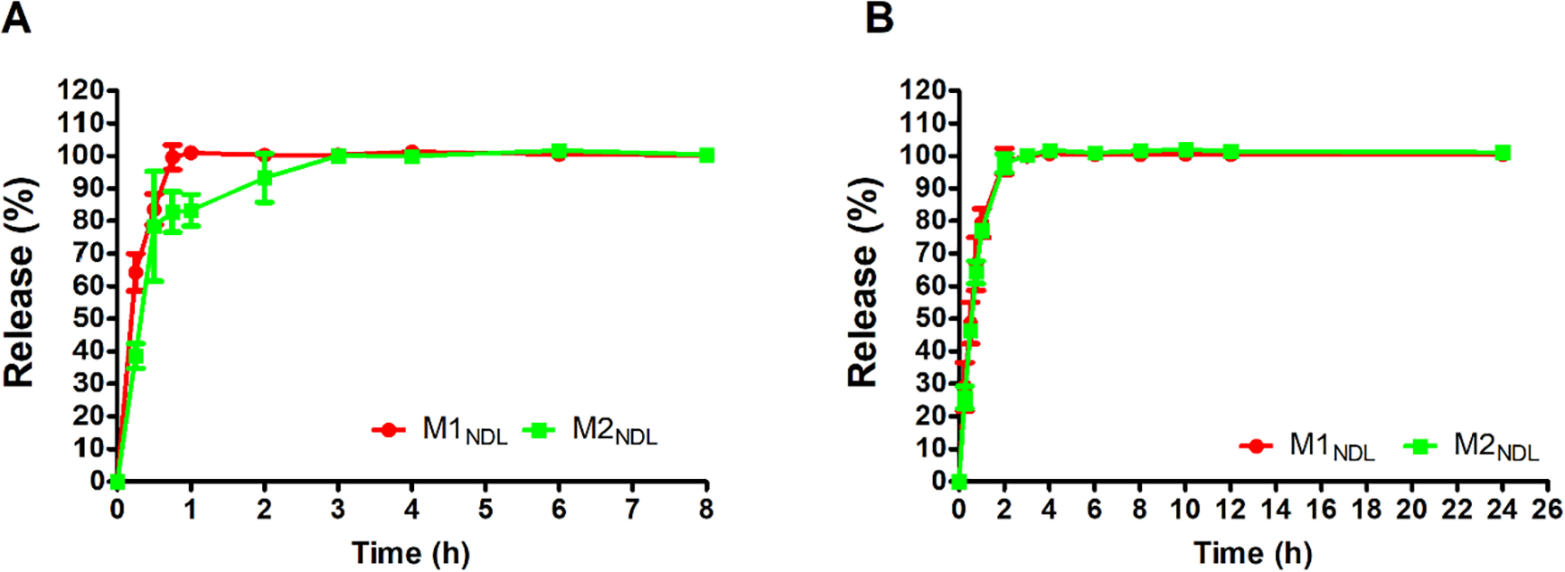
% release of M1_NDL_ and M2_NDL_ formulations in (A) gastric media and (B) intestinal media (±SD).

The calculated difference and similarity factors for pairwise intraformulation comparisons are determined. M1_NDL_ compared to M2_NDL_ showed statistically different release profiles (*f*_1_: 21, *f*_2_: 30); in addition, M2_NDL_ compared to M1_NDL_ was found to be significantly different (*f*_1_: 22, *f*_2_: 31).

### 3.7 Permeability Studies in the Caco-2 Cell Monolayer of Nadolol

Formulation-related toxicity toward Caco-2 cells was evaluated using an MTT test, and as shown in Figure 4, the observed viabilities after exposure remained >95%.^78^

**Figure 4:**
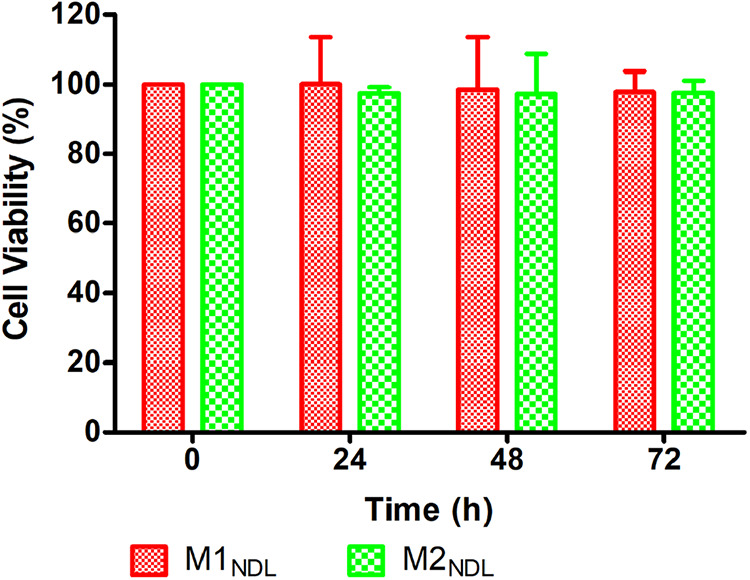
Cell viability % graph of optimum formulations containing NDL (±SD).

During the radiolabeling phase, every formulation received Tc-99m, with subsequent radioactivity readouts obtained on a γ-counter. The NDL transport profile stratified by formulation and direction is shown in Figure 5. Across Caco-2 cells, permeability was significantly higher for the Tc-99m formulations in both A→B and B→A flux studies.

**Figure 5:**
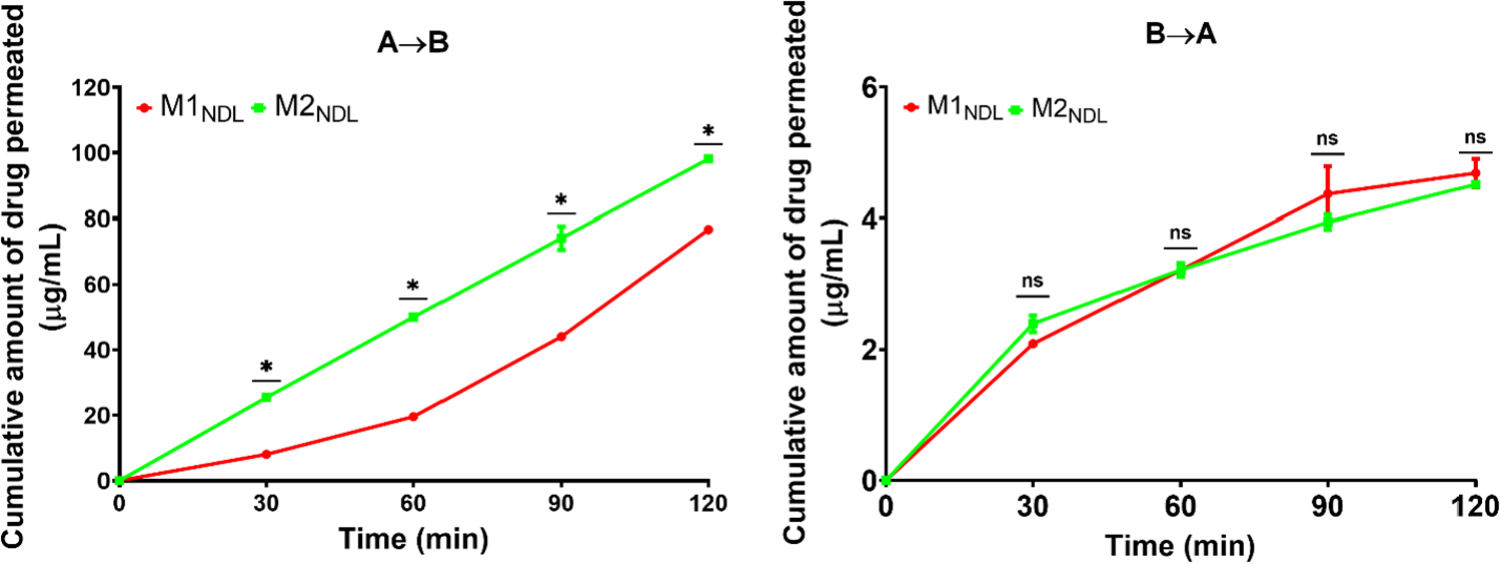
Data on NDL transition from apical to basolateral (A→B) and basolateral to apical (B→A). * for *p* < 0.05; ns for nonsignificant differences.

Surfactant molecules accumulate at the surfaces of emulsion droplets to create an interfacial film, thereby diminishing the interfacial free energy. We observed that loading NDL into a microemulsion significantly increased NDL permeability versus the solution form. The high surfactant/cosurfactant content of microemulsions may also promote permeability by disrupting the cell membrane.

For apical-to-basolateral (A→B) transport, a paired *t* test demonstrated a statistically significant increase in permeability for M2_NDL_ compared to M1_NDL_ (p = 0.0235). The mean difference was 19.86 (95% CI: 4.40–35.33), indicating enhanced absorptive transport.

In contrast, for basolateral-to-apical (B→A) transport, no statistically significant difference was observed between M2_NDL_ and M1_NDL_ (*p* = 0.6562). The mean difference was −0.0577 (95% CI: −0.3911 to 0.2757), suggesting comparable efflux profiles between the two formulations.

In order to overcome the challenge of limited oral bioavailability, microemulsions serve as advanced drug delivery systems that enhance the gastrointestinal absorption of poorly bioavailable compounds. Typically, their formulation comprises an oil phase, a surfactant, a cosurfactant, and an aqueous phase.

In post-transport testing, Caco-2 monolayers underwent a single evaluation based on TEER (Figure 6), recorded immediately before and after each run. Because TEER reflects epithelial barrier performance and is widely adopted as a cytotoxicity criterion, data obtained with the NDL formulation demonstrated no meaningful change over 2 h for either transport vector (A→B, B→A).

**Figure 6:**
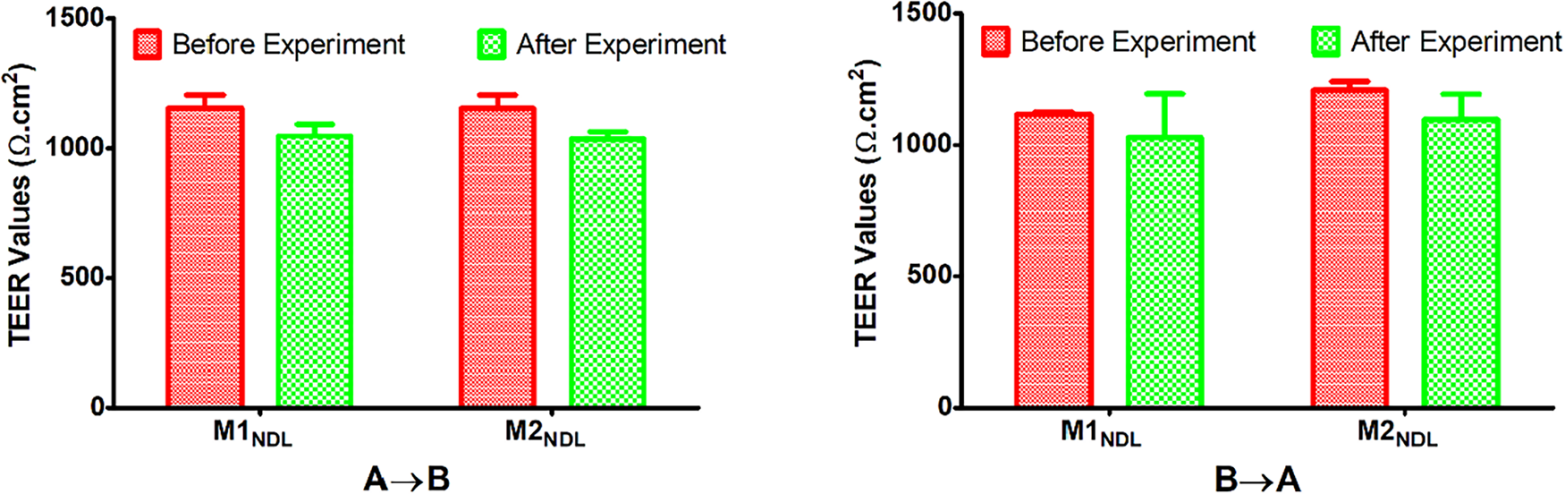
Graph of TEER values measured before and after the experiment in studies of NDL transition from apical to basolateral (A→B) and basolateral to apical (B→A) (±SD).

Even with modest declines in the TEER recorded across the experiments, no structural compromise of the monolayer occurred; accordingly, Caco-2 cultures remained viable at the end of the permeability studies.

### 3.8 SPIP Study

The aim of the study was to evaluate intestinal permeability in rats with SPIP and to predict the absorption of NDL in humans.^79^ The SPIP study, which is one of the FDA-recommended methods, was conducted for the estimation of *in vivo* oral bioavailability in humans. PR is a nonabsorbable marker. It has been used to determine the extent of water exchange throughout the gut and is used to correct the results of inconsistent permeability due to leakages in the intestine caused by various reasons. Adjustments were made using the inlet and outlet concentrations of PR. NDL (high solution and low permeability) is classified as Class III. MPT is classified in Class I (high solution and higher permeability).^49^ The reference drug, MPT, was used to compare the permeability of the NDL.

NDL was detected at 206 nm and the retention time was approximately 3.1 min. The response signal linearity ranges from 20 to 200 (μg/mL). The *R* ^2^ value was determined as 0.9975. Precision (reproducibility and reproducibility), accuracy, and the recovery coefficient of variation were below 2%. LOD and LOQ values were determined as 0.037 and 0.113. It has also been validated for selectivity, sensitivity, linearity, precision, accuracy, stability in MPT and PR.

In the control group, NDL was compared with MPT for *P*_eff_ value determination. NDL and MPT permeabilities were determined as 0.023 ± 0.012 × 10^–4^ cm/s and 0.384 ± 0.032 × 10^–4^ cm/s, respectively, in the study conducted in the control group. It was observed that the permeability of the NDL solution was significantly low. The effect of M2_NDL_ on the active substance permeation, as well as the effect of the blank formulation on the active substance permeation, was evaluated. The M2_NDL_ formulation (*P*_eff_: 0.134 ± 0.042 × 10^–4^ cm/s) was found to have 5.826 times higher NDL permeation than that of the pure NDL solution (*P*_eff_: 0.023 ± 0.012 × 10^–4^ cm/s) (Table 4). The estimated *P*_eff_ and % absorbed active substance fraction (*F*_a_) in humans calculated using the *P*_eff_ value obtained from rats are given in Table 4 and Figure 7, respectively. The possible reason for this significant increase was thought to be the penetration-enhancing components in the formulation loosening the tight junctions in the intestinal apical cells and increasing paracellular permeation. The change in the TEER value measured before and after the *in vitro* release experiment supports the loosening of the tight junctions between the cells.

**Figure 7:**
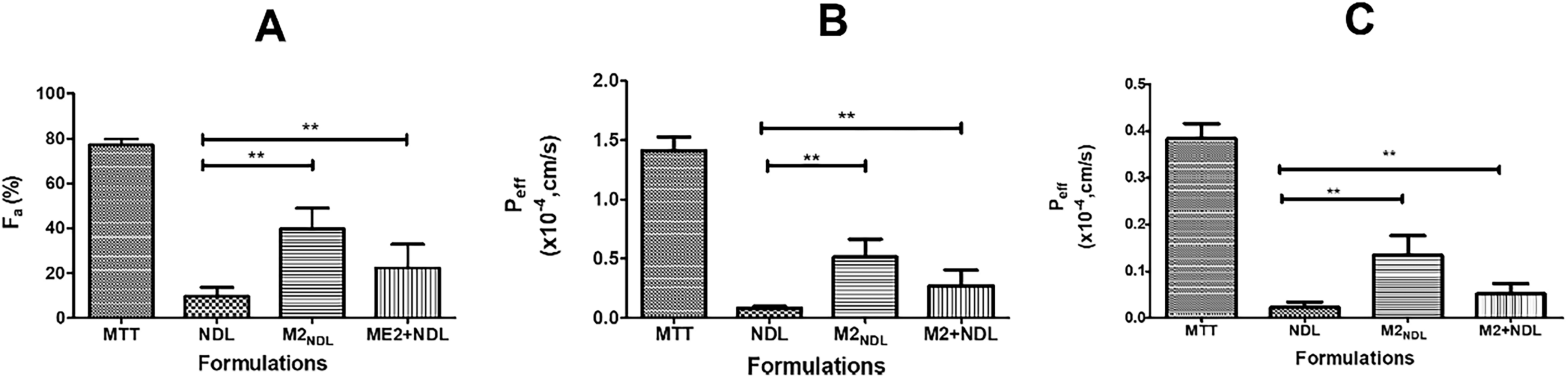
Estimated human *F*_a_ % (A), *P*_eff_ (B), and rat *P*_eff_ (C) values obtained from the SPIP study (±SD).

**Table 4:** Rat *P*_eff_, Estimated Human *P*_eff_, and Estimated Human *P*_eff_ of NDL (*n* = 6)

drug/system	rat *P*_eff_ (±SD) (×10^–4^ cm/s)	estimated human *P*_eff_ (±SD) (×10^–4^ cm/s)	estimated human *F*_a_ (±SD) (10^–2^)
MPT	0.384 ± 0.032	1.413 ± 0.114	77.044 ± 2.757
NDL	0.023 ± 0.012	0.087 ± 0.013	9.771 ± 4.059
M2_NDL_	0.134 ± 0.042	0.514 ± 0.151	39.821 ± 9.202
M2+NDL	0.052 ± 0.022	0.273 ± 0.062	18.180 ± 4.531

Li et al. investigated the effect of microemulsions on the oral bioavailability of metformin hydrochloride (MET), a BCS class III active substance, with a SPIP study. The MET-A microemulsion they developed was composed of 40% oil-phase glyceryl monooleate (GTCC)–Maisine 35-1, 35% surfactant phase Tween 80–Cremophor EL–ethanol mixture, and MET-B was composed of 35% oil phase caprylic/capric triglyceride (GTCC)–Maisine 35-1 and 45% surfactant-phase Tween 80–Cremophor EL–ethanol mixture. In the SPIP study, the permeability of the formulations was investigated in the duodenum, jejunum, and ileum segments of the intestine. In all three segments, the MET solution showed low permeability compared to MET-A and -B. MET-A and -B showed close permeability values. The highest MET permeability was detected in the ileum. In the study, MET, MET-A, and MET-B were administered orally to rats. Considered together with the SPIP study, it has been reported that the ideal formulation improves the oral bioavailability of MET, a BCS class III drug, by increasing lymphatic absorption.^80^

A one-way ANOVA revealed a statistically significant difference among the groups (*p* < 0.0001). Posthoc Tukey’s multiple comparison test showed that M2NDL had significantly higher Peff values compared to NDL (mean difference = 0.1120, 95% CI: 0.0596–0.1644, *p* < 0.0001). No significant difference was observed between NDL and M2+NDL (mean difference = 0.0291, 95% CI: −0.0282–0.0865, *p* = 0.4819). In contrast, M2NDL also differed significantly from M2+NDL (mean difference = 0.0829, 95% CI: 0.0209–0.1448, *p* = 0.0076). All other pairwise comparisons were statistically significant (*p* < 0.0001).

M1 and M2 formulations were developed for the oral delivery of nadolol, a BCS class III drug. M2 was selected for further studies due to its superior performance compared to M1. The improved performance of M2 is attributed to its smaller droplet size, higher HLB value, and the presence of a propylene glycol/ethanol cosolvent system, which enhanced solubilization and interfacial properties. Both formulations exhibited complete *in vitro* drug release, while M2_NDL_ showed more controlled release in simulated gastric conditions. In Caco-2 permeability assays, M2_NDL_ demonstrated higher A→B transport of nadolol than M1_NDL_. Furthermore, SPIP studies revealed that M2_NDL_ achieved a 5.8-fold higher *P*_eff_ in rats compared to a nadolol solution. These findings align with the previous literature reporting that microemulsion systems can significantly enhance the oral absorption of other BCS class III drugs, emphasizing the critical role of droplet size, surfactant HLB, and cosolvent composition in improving oral bioavailability.

Microemulsion and related self-emulsifying drug delivery systems have been explored in the literature to enhance the oral absorption of other BCS class III drugs exhibiting high solubility but low intestinal permeability. For example, fexofenadine, a BCS III antihistamine with limited oral bioavailability, has been formulated as a water-in-oil microemulsion, resulting in significantly enhanced intestinal uptake and relative oral bioavailability compared to conventional sirup, attributed to nanometer-sized droplets and surfactant-mediated permeation enhancement. In this study, the relative bioavailability of the fexofenadine microemulsion was reported to be approximately 3.8-fold higher than that of the commercial formulation in rabbits, with improved Caco 2 permeability *in vitro*.^81^ Likewise, microemulsion approaches applied to other poorly permeable hydrophilic drugs such as famotidine have demonstrated increased intestinal permeability and improved pharmacokinetic profiles, with enhanced permeation attributed to small droplet size, surfactant/cosurfactant effects on the intestinal barrier, and prolonged gastrointestinal residence. These findings underscore the potential of microemulsion systems to overcome permeability limitations inherent to Class III drugs, reinforcing the observation in the current study that an optimized microemulsion formulation can substantially enhance nadolol permeability and *P*_eff_ compared to a simple solution and less optimized formulation.

### 3.9 Storage Stability Studies

The formulation of M2_NDL_ was evaluated for stability determination by examining the droplet size, PDI, ζ-potential, and physical appearance at 5 ± 3 °C (refrigerator), 25 ± 2 °C–60 ± 5% RH (room conditions), and 40 ± 2 °C–75 ± 5% RH (humidity chamber) for 12 months initially and at specific time intervals. No significant changes in droplet size, PDI, ζ-potential, and content were observed (Table 5).

**Table 5:** Results of Storage Stability Study Measurements of the M2_NDL_ Formulation (±SD)

		M2_NDL_		
time (months)	measurements^a^	4 °C	25 °C	40 °C
0	PS (nm)	1.004 ± 0.008	1.004 ± 0.008	1.004 ± 0.008
	PDI	0.291 ± 0.011	0.291 ± 0.011	0.291 ± 0.011
	ZP (mV)	–9.800 ± 0.049	–9.800 ± 0.049	–9.800 ± 0.049
	content quantification	101.173 ± 2.586	101.173 ± 2.586	101.173 ± 2.586
3	PS (nm)	0.435 ± 0.005	0.404 ± 0.025	0.496 ± 0.077
	PDI	0.228 ± 0.029	0.271 ± 0.026	0.351 ± 0.037
	ZP (mV)	–2.360 ± 0.031	–1.280 ± 0.300	–6.520 ± 0.110
	content quantification	98.513 ± 0.587	98.884 ± 0.711	97.358 ± 0.001
6	PS (nm)	0.416 ± 0.019	0.421 ± 0.027	0.385 ± 0.025
	PDI	0.233 ± 0.021	0.304 ± 0.028	0.276 ± 0.053
	ZP (mV)	–3.700 ± 0.050	–1.940 ± 0.870	–2.310 ± 0.710
	content quantification	98.959 ± 0.546	97.938 ± 0.015	97.286 ± 0.047
12	PS (nm)	0.515 ± 0.065	0.443 ± 0.041	0.382 ± 0.006
	PDI	0.356 ± 0.067	0.351 ± 0.066	0.280 ± 0.070
	ZP (mV)	–1.870 ± 0.080	–1.670 ± 0.290	–2.120 ± 0.070
	content quantification	98.798 ± 0.899	97.400 ± 0.006	97.389 ± 0.012

a PS: particle size; PDI: polydispersity index; and ZP: ζ-potential.

## 4 Conclusions

Hypertension is one of the diseases that has been studied extensively since it has been seen worldwide from the past to the present and has a high mortality and morbidity rate. NDL is a nonselective β-blocker approved for the treatment of hypertension. It was loaded into microemulsion drug delivery systems in order to improve the bioavailability of NDL, which is in BCS class III, and to create an alternative to its other forms in the market. In characterization studies, it was determined that the loaded formulation had a low and homogeneous droplet size distribution, pH, and viscosity suitable for oral use. In the *in vitro* release study, the formulations released all of the ingredients. In the permeability study, it increased the permeability without damaging the Caco-2 cells. In the cytotoxicity study, it showed high cell viability, which is an indication of its safety. The SPIP study showed a significant increase in intestinal transit compared to the NDL suspension. The developed microemulsion formulation provided successful results in improving the oral bioavailability of the NDL. Future research could focus on clinical translation, patient compliance, and extending microemulsion-based delivery strategies to other poorly absorbed drugs, building on established formulation optimization and pharmacokinetic findings.

## Data Availability

The data underlying this article will be shared upon reasonable request to the corresponding author. All data in this study are presented in the manuscript.
